# HIV Incidence and Risk Factors in Chinese Young Men Who Have Sex with Men—A Prospective Cohort Study

**DOI:** 10.1371/journal.pone.0097527

**Published:** 2014-05-30

**Authors:** Zhenxin Dong, Jie Xu, Hongbo Zhang, Zhi Dou, Guodong Mi, Yuhua Ruan, Limei Shen, Xiangdong Min, Guanghua Lan, Fan Li, Tian Li, Zhen Ning, Guohui Wu, Min She, Zunyou Wu, for the China National HIV Prevention Study Group

**Affiliations:** 1 School of Public Health, Anhui Medical University, Hefei, Anhui Province, China; 2 National Center for AIDS/STD Control and Prevention, Chinese Center for Disease Control and Prevention, Beijing, China; 3 Guizhou Provincial Center for Disease Control and Prevention, Guizhou, China; 4 Yunnan Provincial Center for Disease Control and Prevention, Yunnan, China; 5 Guangxi Provincial Center for Disease Control and Prevention, Guangxi, China; 6 Xinjiang Center for Disease Control and Prevention, Xinjiang, China; 7 Sichuan Provincial Center for Disease Control and Prevention, Sichuan, China; 8 Shanghai Center for Disease Control and Prevention, Shanghai, China; 9 Chongqing Center for Disease Control and Prevention, Chongqing, China; UNC Project-China, China

## Abstract

**Objectives:**

To assess HIV incidence and its associated risk factors among young men who have sex with men (YMSM) in urban areas, China.

**Design:**

The study used a prospective cohort study design and standard diagnostic tests.

**Methods:**

A twelve-month prospective cohort study was conducted among YMSM (18–25 years old) in 8 large cities in China. The participants were recruited via snowball sampling. A total of 1102 HIV-negative YMSM completed baseline assessment, 878 YMSM participants completed 6-month follow-up, and 902 completed 12-month follow-up. HIV was screened by an enzyme-linked immunosorbent assay and confirmed with Western Blot. Syphilis was screened via rapid plasma reagent and confirmed by treponema pallidum particle agglutination assay.

**Results:**

78 HIV seroconversions were identified within 1168.4 person-year observations yielding an incidence rate of 6.7 per 100 person-years. HIV seroconversion was associated with non-student status (RR = 2.61, 90% CI = 1.3–5.26), low HIV transmission knowledge (RR = 8.87, 90% CI = 2.16–36.43), and syphilis infection (RR = 5.04, 90% CI = 2.57–9.90).

**Conclusions:**

Incidence of HIV among YMSM is high in urban areas of China. Interventions measures are required to contain the HIV epidemic within this population.

## Introduction

The heavy burden of HIV infection among men who have sex with men (MSM) has been demonstrated around world. The HIV prevalence within this subpopulation appears to be rising rapidly and MSM continue to be disproportionately affected by HIV in many international settings [1]. HIV prevalence among MSM in developed countries has been well characterized. For example, MSM accounted for 61% of newly acquired HIV infections in the United States as of 2009 [2], 28% of all new HIV infections in Canada as of 2008 [3], and 72% of HIV infections in Germany as of 2009 [4]. More recently, high prevalence of HIV among MSM in middle and low-income countries has been identified [1]. A review for the studies conducted between 2000 and 2006 estimated the prevalence of HIV among MSM was 17.9% in sub-Saharan Africa, 14.7% among MSM in South and Southeast Asia, 14.9% in central and South America, 25.4% in the Caribbean, and 5.2% in East Asia [1].

A number of studies in China have revealed rapidly increasing rates of HIV infection among MSM [5], [6]. The estimated proportion of newly infected HIV cases attributable to sexual behavior between men increased from 12.2% in 2007 to 32.5% in 2009 and 29.4% in 2011 [7], [8]. Sentinel surveillance reports have documented rising HIV prevalence among MSM in Harbin (1.2% in 2003 to 10.3% in 2011), Chengdu (from 0.6% in 2003 to 16.3% in 2011) [9], and other metropolitan cities in China [10]–[13]. The cross-sectional designs of previous studies limit them to the estimation of HIV prevalence among MSM. Research on HIV incidence and risk factors that predict new infections among MSM in China is needed.

Young MSM (YMSM; between 18–25 years old) have been shown to be a particularly high risk population in many international settings [14]–[17]. Rates of new infection have been consistently high among YMSM in epidemiological studies [18]. Psychological distress and substance use have been associated with increased sexual risk behaviors during adolescence [19]. Due to stigma associated with sexual orientation, YMSM in many regions may lack access to gay-specific HIV prevention education information and positive role models; these factors can increase risk for their acquisition of HIV [20]. Researchers in China have only recently begun to pay attention to HIV infection among YMSM. A study of YMSM in Hefei, China in 2005 found that 87.2% had one or more homosexual sexual partners, only 17.4% used condoms consistently for anal intercourse in the last 6 months, and the YMSM sample had a 1.0% HIV infection rate [21]. Another study among YMSM in Beijing, Harbin, Zhengzhou and Chengdu found that the prevalence of HIV infection and syphilis infection were 6.7% and 8.3% in 2008, respectively [22]. However, the above-mentioned studies examined HIV prevalence among YMSM. A small number of cohort studies have examined HIV incidence in MSM, though few in YMSM. For example, a cohort study of MSM in Beijing reported HIV incidence of 2.6 per 100 person-years in 2006–2007 [23]. Another 6-month prospective cohort study reported the HIV incidence was 5.12 per 100 person-years in 2008 among MSM in Nanjing [24]. In Shenyang, researchers followed up 218 HIV-negative MSM, and found that the HIV incidence was 5.4 per 100 person-years in 2006–2007 [25]. In light of the worldwide HIV infection trends among YMSM, estimates of HIV incidence and associated factors among YMSM in China are needed [26]. The present study aimed to assess the HIV incidence rate and identify risk factors for HIV infection among YMSM in eight large cities in China.

## Methods

### Study Participants

During August 2009 to December 2010, a cohort study was conducted in eight cities of China including: Beijing, Shanghai, Kunming, Guiyang, Chongqing, Chengdu, Urumqi and Nanning. Eligibility criteria of participants were men ages 18–25, who had anal or oral sex with men in the past 6 months, who were tested to be HIV negative at recruitment, and who were willing to participate in 6- and 12-month follow-up surveys as well as complete follow-up HIV testing. Because of the hard reach to the population, we used snowball sampling to recruit participants. In the baseline study, 10 initial seed subjects from each city were recruited. After training of recruitment eligibility, these subjects were given “recruitment coupons” to refer other YMSM in their networks to the study, who were then screened for eligibility and invited to participate if eligible. All subsequent participants were asked to refer other YMSM to this study using the recruitment coupons. Each recruitment coupon had an expiration date, information about the study testing site, and a contact telephone number. The baseline survey was conducted with 1242 YMSM. Of them, 140 (11.3%) were HIV positive at baseline and were excluded due to calculate the rate of new infections. The remaining 1102 YMSM were eligible for the cohort study.

### Procedures

After completing written informed consent, structured questionnaire-based interviews were administered in a private room by health workers from the local Centers for Disease Control and Prevention (CDC). Blood samples were collected from participants for tests of HIV and syphilis infections by health workers. An enzyme-linked immunosorbent assay was used to screen for HIV antibodies (ELISA; InTec PRODUCTS, INC., Xiamen, China) and Western Blot immunoassay (WB; Singapore MP Biomedical Asia Pacific Ltd Singapore, Singapore) was used for HIV-1/2 confirmation if ELISA was positive. All participants received pre-and post-test HIV counseling by CDC workers. Rapid Plasma Reagent (RPR; Kinghawk Pharmaceutical Co., Ltd., Beijing, China) was used for syphilis screening and Treponema pallidum particle agglutination assay (TPPA; Fujirebio Inc., Tokyo, Japan) was used for confirming syphilis infection. Individuals who were HIV positive or syphilis infected were referred to HIV/STD care clinics for appropriate services. Participants provided contact information and were informed about the 6- and 12-month follow-up assessments.

We conducted 6-month follow-up assessments with 878 YMSM and 12-month follow-up assessments with 902 YMSM. Follow-up assessments involved identical procedures including the behavioral risk questionnaire and HIV test.

### Ethics Statement

This study, including design, recruitment, consent, and assessment procedures, was reviewed and approved by the Institutional Review Board of the Chinese National Center for AIDS/STD Control and Prevention, Chinese Center for Disease Control and Prevention. Written informed consent was obtained from all participants. Each participant received 50 RMB (8 USD) and 30 condoms as incentive for participation in baseline and follow-up assessments.

### Measures

Questionnaire-based interviews were administrated to assess socio-demographic characteristics (marital status, residence, education, occupation, sexual orientation), behavioral information (in the past 6 months: sex with male partners, number of male sex partners, frequency of condom use during anal sex with men, sex with male partners for money, frequency of condom use in commercial sex), and any STD diagnosis. Knowledge related to HIV transmission was measured by a 8-question measure (sample question: “Do you think you can be infected with HIV by having a meal with HIV-positive people?”; response options were “Yes”, “No”, “I don’t know”). Knowledge scores were created by counting the number of correct responses; “I don’t know” was coded as an incorrect response.

### Data Analysis

EpiData 3.0 (The EpiData Association, Odense, Denmark) software was used to input the original data and SPSS 10.01 (SPSS Inc Chicago, IL) was used for data analysis. Descriptive analyses were conducted to describe socio-demographic characteristics of the sample. Chi-square tests were conducted to examine differences in socio-demographic characteristics between baseline study and follow-up studies. HIV incidence was calculated by using the number of seroconversions within the follow-up period as the numerator and the cohort’s total number of person-year (PY) exposure to the risk of HIV transmission as the denominator. Cox regression analysis was conducted to identify the risk factors for HIV seroconversion. Independent variables were entered into the multivariate Cox regression models in 3 blocks including socio-demographic characteristics (block 1), HIV/AIDS related knowledge and sexual behaviors (block 2), and syphilis and STD infection (block 3).

## Results

### Socio-demographic Characteristics

Socio-demographic characteristics of the respondents are described in Table 1. A total of 1102 YMSM enrolled in the cohort study, and the median age was (22.03±2.07) years with a range from 18 to 25 years. Of 1102 YMSM, 6.9% were currently married and 56% did not have a residence card in the study city, indicating they were migrants. Approximately two-thirds (61.9%) had a college or higher level of education. One-third (33.9%) were students. The majority (66.8%) identified themselves as homosexual, 29.0% identified as bisexual, and 4.2% identified as heterosexual or unsure.

**Table 1:** Socio-demographic Characteristics of YMSM cohort participants in Baseline and Follow-up.

Characteristics	baseline(N = 1102)	6-month follow-up(N = 878)			12-month follow-up(N = 902)		
	n (%)	n (%)	*χ^2^*	*P*	n (%)	*χ^2^*	*P*
**Marital status**							
Single	1026 (93.1)	812 (92.5)	0.283	0.595	837 (92.8)	0.073	0.787
Married/cohabit	76 (6.9)	66 (7.5)			65 (7.2)		
**Local resident**							
Yes	485 (44.0)	394 (44.9)	0.148	0.701	392 (43.5)	0.061	0.804
No	617 (56.0)	484 (55.1)			510 (56.5)		
**Education**							
Junior high school or less	121 (11.0)	80 (9.1)	4.366	0.113	103 (11.4)	0.135	0.935
Senior high school	299 (27.1)	216 (24.6)			247 (27.4)		
Some college or higher	682 (61.9)	582 (66.3)			552 (61.2)		
**Occupation**							
Student	374 (33.9)	309 (35.2)	2.075	0.722	292 (32.4)	0.740	0.946
civil servants/White-collar	186 (16.9)	159 (18.1)			157 (17.4)		
Waiter	404 (36.7)	295 (33.6)			343 (38.0)		
Workman/Farmer	46 (4.2)	38 (4.3)			37 (4.1)		
Unemployed	92 (8.3)	77 (8.8)			73 (8.1)		
**Sexual orientation**							
Homosexual	736 (66.8)	603 (68.7)	0.925	0.630	598 (66.3)	0.105	0.949
Bisexual	320 (29.0)	243 (27.7)			264 (29.3)		
Heterosexual/other	46 (4.2)	32 (3.6)			40 (4.4)		

Of 1102 YMSM in the baseline survey, 878 (79.7%) participated in the 6-month follow-up. Nine hundred and two YMSM (81.9%) completed assessment at the 12-month follow-up. There were no significant differences on socio-demographic characteristics between participants who completed the baseline and 6- or 12-month follow-up visits (Table 1).

### HIV Seroconversions and Incidence Rate among YMSM

Table 2 describes the HIV incidence at 6- and 12-month follow-ups. Of 878 participants who completing the 6-month follow-up, 39 were infected with HIV. Of 902 participants who completed the 12-month follow-up, 39 were infected with HIV. Overall, 1102 participants were followed for 1168.40 person-years, during which 78 HIV seroconversions occurred. The incidence rate was 6.7 per 100 person-years (PY) during one year of follow-up. HIV incidence rate during the 12-month follow-up varied by city. In descending order, t HIV incidence rates were 18.9/100 PY in Guiyang, 10.6/100 PY in Beijing, 5.6/100 PY in Shanghai, 5.3/100 PY in Kunming, 4.9/100 PY in Chongqing, 4.8/100 PY in Nanning, 4.3/100 PY in Urumqi and 3.9/100 PY in Chengdu (data not shown in tables).

**Table 2:** Sociodemographic characteristics associated with HIV incidence of YMSM cohort participants.

Factors	Incidence of HIV in one year						
	No. of seroconversionsat 6-month follow-up	No. of seroconversionsat 12-month follow-up	No. of seroconversionsin one year	Person-year		Incidence rate (/100 PY)	
**Education**							
Junior high school or less	3	5	8	127.18		6.4	
Senior high school	13	15	28	321.81		8.7	
Some college or higher	23	19	42	719.41		5.8	
**Student**							
Yes	12	5	17	400.98		4.2	
No	27	34	61	767.42		7.9	
**Age**							
18∼20	13	12	25	310.61		8.0	
21∼25	26	27	53	857.79		6.2	
**Local resident**							
Yes	19	15	34	534.68		6.4	
No	20	24	44	633.72		6.9	
**Homosexual**							
Yes	24	24	48	777.84		6.2	
No	15	15	30	390.56		7.7	
**Total**	39	39	78	1168.40		6.7	

### Risk Factors for HIV Seroconversion in One Year

We used multivariate Cox regression models to examine associations of risk factors with HIV seroconversion in one year (Table 3). In the first model, HIV seroconversion was associated with non-student status (RR = 2.69, 90% CI = 1.35–5.39). In the second model, HIV seroconversion was associated with lower levels of HIV/AIDS transmission knowledge (RR = 8.66, 90% CI = 2.24–33.56) and non-student status (RR = 2.83, 90% CI = 1.41–5.69). In the third model, HIV seroconversion was associated with syphilis infection (RR = 5.04, 90% CI = 2.57–9.90), lower levels of HIV/AIDS transmission knowledge (RR = 8.87, 90% CI = 2.16–36.43), and non-student status (RR = 2.61, 90% CI = 1.30–5.26).

**Table 3:** Factors associated with HIV Seroconversions in 12-month follow-up study among YMSM in Cox regression analysis.

Factors	Block 1 *RR* (90% *CI*)	Block 2 *RR* (90% *CI*)	Block 3 *RR* (90% *CI*)
**Socio-demographic characteristics**			
** Age** a	1.60 (0.87–2.92)	1.45 (0.79–2.68)	1.22 (0.65–2.29)
** Marital status** a	1.10 (0.46–2.64)	0.96 (0.38–2.41)	0.97 (0.38–2.45)
** Local resident** a	1.43 (0.86–2.38)	1.37 (0.81–2.32)	1.27 (0.75–2.18)
** Education level**			
Some college or higher	1	1	1
Junior high school or less	0.87 (0.37–2.06)	0.87 (0.36–2.09)	0.74 (0.30–1.77)
Senior high school	1.17 (0.65–2.11)	1.18 (0.65–2.14)	1.22 (0.67–2.24)
** Student** a	2.69 (1.35–5.39)*	2.83 (1.41–5.69)*	2.61 (1.30–5.26)*
** Homosexual** a	1.39 (0.85–2.27)	1.38 (0.84–2.27)	1.46 (0.88–2.41)
**Knowledge and sex behaviors**			
HIV/AIDS knowledge^a^		8.66 (2.24–33.56)*	8.87 (2.16–36.43)*
Had anal sex with menin the past 6 months a		0.80 (0.34–1.86)	0.87 (0.38–2.02)
Male sexual partners for morethan 2 people in the past 6 months^a^		1.11 (0.65–1.90)	1.04 (0.61–1.78)
Consistent condom use with malesex partners in the past 6 months^a^		1.36 (0.81–2.29)	1.30 (0.77–2.19)
Being paid by a man for sexin the past 6 months^a^		1.54 (0.41–5.84)	1.72 (0.43–6.84)
Consistent condom use whenbe paid for sex in the past 6 months^a^		0.00 (0.00–6.58*10^189^)	0.00 (0.00–2.93*10^270^)
**Syphilis and STD**			
Had specific symptoms ofSTD infections in the past 12 months^a^			1.74 (0.89–3.37)
Syphilis infection^a^			5.04 (2.57–9.90)*

*Denotes significance at *P*<0.10 level.

a Dichotomous variables: Age: 0 = 21∼25 years, 1 = 18∼20 years; Married: 0 = no, 1 = yes; Local resident: 0 = yes, 1 = no; Student: 0 = yes, 1 = no; Homosexual: 0 = yes, 1 = no; HIV/AIDS knowledge: 0 = know, 1 = unacquaintance (know: scores of HIV/AIDS knowledge = 6–8, unacquaintance: scores of HIV/AIDS knowledge = 0–5); Had anal sex with men: 0 = no, 1 = yes; Male sexual partners for more than 2: 0 = no, 1 = yes; Consistent condom use with male sex partners: 0 = yes, 1 = no; Being paid by a man for sex: 0 = no, 1 = yes; Consistent condom use when paid for sex: 0 = no, 1 = yes; Had specific symptoms of STD infections in the past 12 months: 0 = no, 1 = yes; Syphilis infection: 0 = no, 1 = yes.

## Discussion

This cohort study brings attention to YMSM in China as a sub-group of MSM at high risk for HIV infection. The present study reveals that the HIV incidence rate was 6.7 per 100 person-years among YMSM. In addition, the study shows that YMSM with non-students status, low HIV/AIDS transmission knowledge, and syphilis infection were more likely to become infected with HIV. These findings propose some measures for developing HIV intervention and prevention programs targeting YMSM. In particular, prevention and intervention efforts should target non-student YMSM populations in urban China to improve their HIV/AIDS transmission knowledge, and encourage them to seek testing and treatment for syphilis.

From February 2008 to September 2009, a nationwide cross-sectional survey was conducted in 61 cities in China reported that the overall prevalence of HIV was 4.9% among MSM. In that survey, 43.3% of participants were 18–24 year-old MSM, and HIV prevalence in this subgroup was 3.8% [27], which is much lower than the baseline prevalence observed here (11.3%, data were not shown). The difference between study findings might be because HIV prevalence was greater than 3% in the eight cities included in the current study. Apart from the higher HIV prevalence in this study, the HIV incidence rate among the YMSM population is much higher than estimates reported in regions elsewhere, including those samples in Ontario, Canada (generally MSM: 1.39/100 PY in 1999) [28] and Bangkok, Thailand (5.9/100 PY in 2006–2010) [16]. The HIV incidence rate in this study is also higher than estimates derived from MSM cohorts in China followed between 2006 and 2007 (2.6/100 PY in 2006–2007) in Beijing [23], [29], Shenyang [25] and Nanjing [24]. However, these prior cohort studies in China included older MSM, with a mean age greater than 27 years. Thus, the current study indicates that YMSM are at elevated HIV risk and require more intervention efforts.

Data in the current study indicates that YMSM who were non-students status were more likely to be infected HIV compared to student YMSM (7.9/100 PY vs.4.2/100 PY). These findings are similar to a study conducted in Chongqing province in 2009, which found that compared to students, non-students MSM have higher HIV infection prevalence (4.4% versus 20.9%) [30]. This findings is consistent with a previous study which found that HIV infection was associated with lower education level [22]. Findings might suggest that MSM enrolled in school or university maybe be in a relatively safe environment or have higher HIV/AIDS-related knowledge. However, recent studies found risk homosexual behaviors such as casual or commercial sex and unprotected sex have been increasingly common among Chinese college students [31]. Thus, future studies should pay attention to HIV risk factors among YMSM students.

Higher HIV/AIDS-related knowledge was associated with more consistent use of condoms with primary and commercial sex partners [32]. A study conducted in Liaoning province indicates that risky sexual behaviors might be indirectly reduced by improving HIV/AIDS-related knowledge [33]. In the present study, we also found that lower levels of HIV/AIDS-related knowledge are important risk factors for HIV infection. Therefore, in the future we should develop intervention studies to improve HIV/AIDS-related knowledge among YMSM. Our study also found that syphilis can increase the risk of HIV infection was consistent with earlier studies. Syphilis facilitates HIV transmission due to ulcers that disrupt the epithelial and mucosal barriers as well as inflammation that increase the HIV virus recruitment of CD4 cells, thereby facilitating and increasing the probability of HIV transmission [34]. A cross–sectional study in Henan showed that MSM infected with syphilis were nearly 5 times more likely to be HIV positive [35]. In India, a cohort study found that participants within 6 months of syphilis infection were 4.44 times more likely to be HIV positive [36].

There are several limitations to this research. Firstly, due to the hard-to-reach nature of the targeted population, the snowball sampling method might not have recruited a representative sample. Moreover, because many MSM in China do not choose to disclose their sexual orientation or might self-identify as heterosexual, this sample might be affected by selection bias. Secondly, non-attendance at follow-up assessments might have affected the HIV incidence rate estimates. However, we believe that this bias might be limited because of the reasonably high follow-up rate (81.9%) and the similarity between those who failed to attend follow-up and those who remained in the cohort. Third, due to the brief nature of the questionnaire, we were unable to examine other potentially important risk factors for HIV infection including alcohol and other drug use, sex with women, health and prevention services utilization, and specific contexts of risk behavior, which need further investigations. Finally, due to the different economic and cultural features in different cities, caution is need when grouping findings across cities to calculate the whole seroconversion rate.

Study strengths included the novelty of addressing the subpopulation of YMSM and assessing HIV incidence in eight large cities in China. This study provides evidence for the high HIV incidence rate among YMSM in China and draws attention to younger MSM as a population in need of urgent public health intervention. Future research is needed to explore the underlying psychological, social, economic and structural factors related to these high HIV risk behaviors among YMSM. Efforts to reduce risk behavior and improve preventative strategies such as HIV/STI testing and treatment are important steps in order to reduce HIV transmission among and from this special group. Future efforts must be attentive to cultural factors, such as stigma and secrecy about same-sex behaviors, which may shape the implementation feasibility and acceptability of HIV interventions for YMSM in China.
